# Remodeling of the brain immune microenvironment in Alzheimer’s disease: interactions among microglia, astrocytes, complement, and the neurovascular unit

**DOI:** 10.3389/fimmu.2026.1956600

**Published:** 2026-09-28

**Authors:** Tong Wang, Lu Wang, Jianing Cao, Yi Zhao, Zongling He, Fengmei Lu

**Affiliations:** The Clinical Hospital of Chengdu Brain Science Institute, MOE (Ministry of Education) Key Lab for Neuroinformation, University of Electronic Science and Technology of China, Chengdu, China

**Keywords:** Alzheimer’s disease, astrocytes, complement system, microglia, precision stratification

## Abstract

Alzheimer’s disease is defined by amyloid-β deposition and tau pathology, yet comparable pathological burdens do not necessarily correspond to similar cognitive trajectories, regional patterns of injury, or treatment responses. This discordance suggests that the cellular and tissue microenvironment may materially shape disease progression. Genetic studies, single-cell and spatial omics, and human-derived models further indicate that microglia, astrocytes, the complement system, and the neurovascular unit undergo coordinated remodeling during the preclinical phase of AD and influence pathological spread, synaptic injury, and neurodegeneration through intercellular interactions. Remodeling of the brain immune microenvironment is regionally specific, stage dependent, and heterogeneous across cellular states; conventional pro-inflammatory/anti-inflammatory or activated/resting dichotomies therefore fail to capture its dynamics. This review examines the remodeling of the AD brain immune microenvironment through interactions among microglia, astrocytes, complement, and the neurovascular unit, with emphasis on their roles in Aβ and tau pathology, synaptic injury, lipid homeostasis, and vascular dysfunction, and considers the interpretive limits of single-cell and spatial-omics evidence. Current evidence supports a dynamically reconfigured system rather than a uniformly escalating inflammatory response. Therapeutic strategies should move beyond broad anti-inflammatory approaches to preserve effective pathological clearance within appropriate disease windows, limit maladaptive synaptic injury, and restore glial and neurovascular homeostasis.

## 1 Introduction

The pathological process of Alzheimer’s disease (AD) may begin more than a decade, and sometimes several decades, before clinical symptoms emerge, progressing from molecular abnormalities to neural-circuit disruption and widespread neurodegeneration. Amyloid-β (Aβ) deposition and tau pathology are central to AD diagnosis and staging and remain the principal targets of disease-modifying therapies. However, similar Aβ and tau burdens do not necessarily produce comparable cognitive trajectories, regional injury, or treatment responses (1–3), suggesting that the cellular and tissue context in which pathological proteins accumulate may substantially influence disease progression.

Glial cells in different brain regions can follow distinct state trajectories, and spatial factors—including proximity to plaques, vascular location, white-matter injury, and local tau burden—can shape their functional states. AD therefore appears to involve persistent remodeling of local brain immune microenvironments rather than dysfunction of a single cell type (4–7). Microglia mediate pathological sensing and phagocytosis; astrocytes maintain neurotransmitter and metabolic homeostasis; complement recognizes and opsonizes abnormal structures; and the blood-brain barrier (BBB) and neurovascular unit regulate molecular exchange, perfusion, and immune trafficking (8–11).

Remodeling of the brain immune microenvironment precedes widespread neuronal loss and evolves across the disease course. Following Aβ deposition, distinct local ecosystems develop around plaques and vessels. As tau pathology spreads, glial responses increasingly involve synaptic tagging, antigen presentation, extracellular-matrix remodeling, and metabolic dysfunction (1, 7, 12). Aβ and tau can therefore reshape the brain immune milieu, while the remodeled cellular and vascular networks may in turn alter the clearance and propagation of these pathological proteins.

A central challenge is to distinguish compensatory responses from processes that directly contribute to tissue injury within highly heterogeneous and dynamic cellular states. Binary frameworks such as activated versus resting or pro-inflammatory versus anti-inflammatory do not capture state continua, regional variation, or disease-stage dependence. Moreover, transcriptomic association and spatial colocalization do not establish function or causal direction. Candidate cellular states and signaling pathways therefore require convergent validation across human brain tissue, longitudinal biomarkers, experimentally tractable human-derived systems, and cell-specific perturbation studies (1, 7, 12, 13).

This review focuses on the dynamic remodeling of the AD brain immune microenvironment, organized around interactions among microglia, astrocytes, the complement system, and the neurovascular unit (As illustrated in **Figure 1**). We examine their roles in Aβ and tau pathology, synaptic injury, lipid metabolism, and vascular dysfunction, and consider patient stratification, target selection, and combination therapies across disease stage, brain region, APOE genotype, and cellular state.

**Figure 1:**
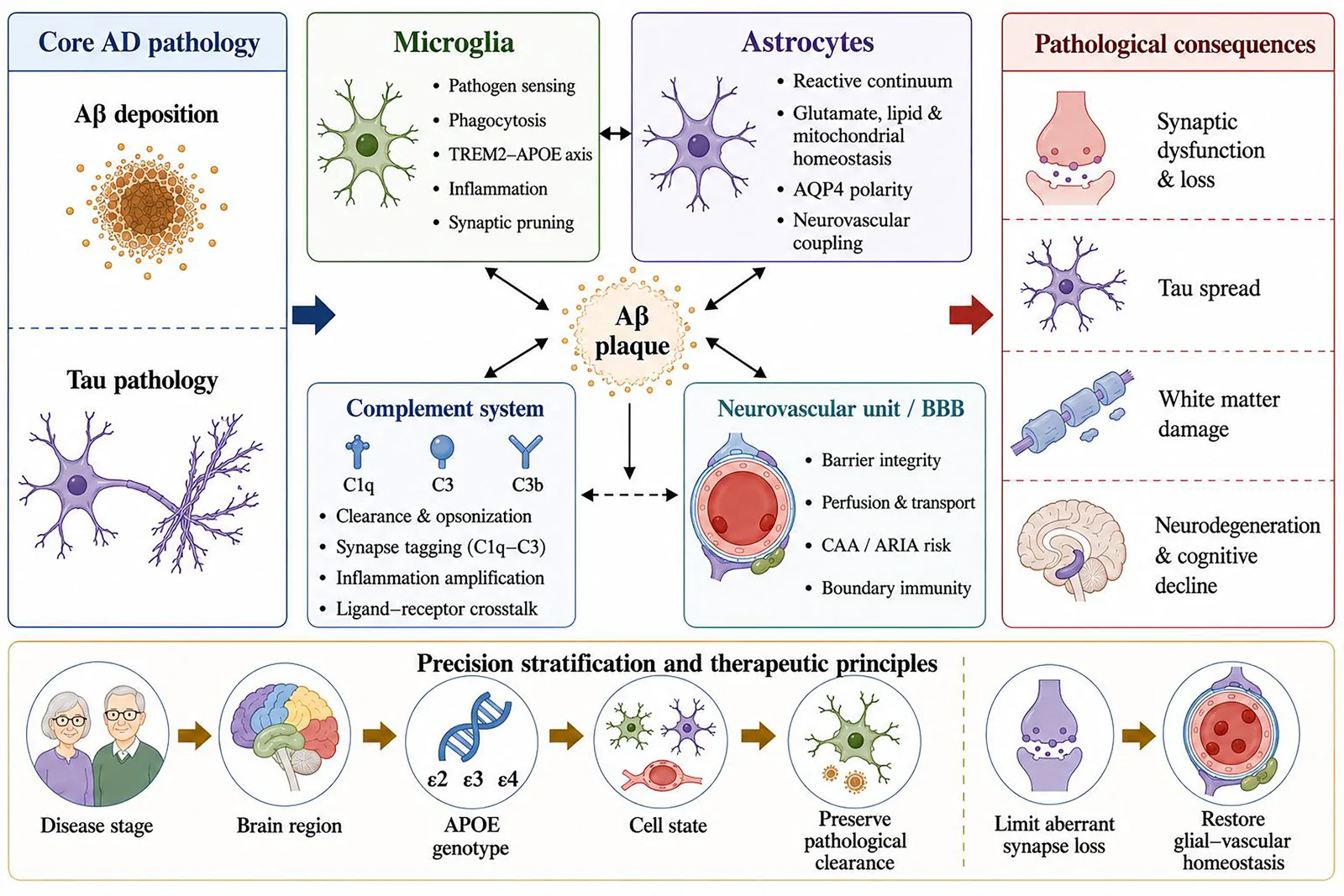
Dynamic remodeling of the brain immune microenvironment in Alzheimer’s disease. Aβ deposition and tau pathology drive stage-dependent and region-specific changes in microglia, astrocytes, the complement system, and the neurovascular unit, thereby influencing pathological protein clearance and spread, synaptic homeostasis, metabolic balance, and vascular function. These processes are shaped by disease stage, brain region, APOE genotype, and cellular state, supporting a shift from broad anti-inflammatory approaches toward biomarker-guided, stratified interventions.

## 2 Literature search and evidence appraisal

We conducted a search in PubMed/MEDLINE up to May 1, 2026. We screened the references of key reviews and original studies. The search keywords included Alzheimer’s disease, amyloid protein, tau protein, neurodegenerative lesions or cognitive decline, as well as terms related to microglia, astrocytes, complement, blood-brain barrier, neurovascular units, bronchopulmonary immune, single-cell or spatial omics, biomarkers, and treatment methods.

The included evidence includes peer-reviewed English human genetic studies, autopsies, cerebrospinal fluid or blood biomarkers, neuroimaging, and intervention studies, as well as animal mechanism studies, traditional *in vitro* experiments, induced pluripotent stem cell systems, organoids, and human-glia cell chimeric models. Original studies with mechanistic and translational significance were given priority; authoritative reviews were used to provide background information. This review is a structured narrative review. In the full text, the observational evidence from humans is clearly distinguished from the evidence from humans, animals, and *in vitro* experiments.

## 3 Organizing principles of the brain immune microenvironment

### 3.1 State continua and spatial compartmentalization

A defining feature of the brain immune microenvironment is the continuity of cellular states. Microglia and astrocytes do not switch between discrete resting and activated states; rather, they transition dynamically among homeostatic maintenance, stress adaptation, antigen presentation, and ageing-related programs (As summarized in **Table 1**) (4–6, 14, 15). Although M1/M2 and A1/A2 classifications remain useful in selected experimental settings, they do not adequately represent cellular heterogeneity in the human AD brain. Spatial context further shapes cell state: local Aβ or tau burden, myelin debris, proximity to vessels, and the extracellular matrix can all alter glial transcriptional profiles and phagocytic function (5, 6, 13, 16–18).

**Table 1:** Representative cellular states in the AD brain immune microenvironment and their functional implications.

Cell/state	Representative markers or pathways	Principal functions	Potential consequences in AD	Evidence and limitations
Homeostatic microglia	P2RY12, TMEM119, SALL1, CX3CR1	Surveillance, synaptic homeostasis, debris clearance	Reduced homeostatic-gene expression indicates microenvironmental stress	Human postmortem and single-nucleus evidence; descriptive and affected by postmortem interval, RNA quality, and nuclear-RNA bias (5, 6)
Plaque-associated/DAM-like microglia	TREM2, APOE, LPL, CST7, CLEC7A	Plaque containment, lipid handling, phagocytosis	May restrict Aβ spread early; chronic states may develop metabolic mismatch	State first defined in mouse models; human signatures overlap only partially and do not imply identical function (20, 33, 34)
Interferon-responsive microglia	IFITM3, ISG15, STAT1, MHC-I	Antiviral responses, antigen presentation	Chronic states may promote C3 expression, chemotaxis, and synaptic injury	Predominantly mouse and ex-vivo mechanistic evidence; corresponding human states are low-abundance and spatially variable (34, 35)
Lipid-droplet-enriched microglia	ACSL1, PLIN2, lipid/lysosomal programs	Lipid buffering and adaptation to phagocytic load	May release neurotoxic lipid factors in an APOE4 context	Human postmortem and iPSC evidence with mechanistic support from models; toxicity depends on genotype and lipid context (36–38)
Reactive astrocytes	GFAP, VIM, SERPINA3, C3, ECM programs	Barrier support; neurotransmitter and metabolic regulation	May clear Aβ but can also amplify complement activity and excitotoxicity	A1/A2 terminology derives from acute experimental models and does not adequately represent human AD astrocyte states (39, 40)
Vascular-associated cell states	CLDN5, PDGFRβ, VCAM1, MMP9	Barrier function, blood flow, and molecular transport	Leakage, peripheral immune recruitment, and reduced Aβ clearance	Human imaging, postmortem, and single-cell evidence is mainly observational; causal mechanisms are partly model-derived (41, 42)

### 3.2 Stage dependence and load-capacity mismatch

The effects of an immune response are not fixed across the disease course. In amyloid and tau mouse models, early plaque containment, debris removal, complement-mediated opsonization, and barrier maintenance can restrict local pathological spread, whereas increasing substrate burden can exceed lysosomal degradation, cholesterol efflux, and mitochondrial capacity and promote lipid-droplet accumulation, inflammasome signaling, maladaptive synaptic clearance, and impaired repair (19–24). Human postmortem, imaging, and fluid-biomarker studies are consistent with stage-related changes in glial and vascular responses, but their cross-sectional associations do not establish the temporal sequence inferred from experimental models. We therefore use load-capacity mismatch as an integrative interpretation, not as a clinically validated disease law: when several processing pathways approach failure simultaneously, local compensation may become unstable and self-reinforcing.

### 3.3 Disease stage, brain region, genotype, and comorbidity jointly shape heterogeneity

Brain immune responses in AD are jointly shaped by disease stage, anatomical region, lesion proximity, and individual background. The preclinical Aβ phase, combined Aβ and tau pathology, and widespread neurodegeneration differ in their dominant loads and likely immune bottlenecks (5, 6). Even within one region, lesion-adjacent and distant cells may follow different trajectories (25). APOE genotype, age, sex, ancestry, cerebrovascular disease, and mixed pathologies further modify these responses. APOE4 influences microglial lipid handling, astrocytic lipoprotein transport, and neurovascular integrity, with effects that vary by sex and ancestry (26–29). Ageing reduces lysosomal and mitochondrial reserve, AQP4 polarity, and vascular support (30–32). Cerebral amyloid angiopathy, small-vessel disease, Lewy body or TDP-43 pathology, metabolic disease, infection, sleep disturbance, and prior treatment can therefore alter both biomarker interpretation and therapeutic risk.

## 4 Microglia: from pathological sensing to phagocytic and metabolic mismatch

### 4.1 State spectra and functional interpretation

Microglia arise from yolk-sac myeloid progenitors and undergo long-term self-renewal in the adult brain (43). Their homeostatic state depends on signals including TGFβ, CSF1/IL-34, and CD200-CD200R, which maintain expression of P2RY12, TMEM119, and CX3CR1. Highly ramified processes continuously survey synaptic activity, extracellular ATP, lipids, and damage-associated signals (44–46). Early in AD, local Aβ, dystrophic neurites, and perivascular protein deposits disrupt homeostasis, prompting microglial recruitment to plaques and formation of a cellular barrier (47, 48).

Disease-associated microglia (DAM) and the related MGnD designation were originally defined through transcriptomic and perturbation studies in mouse models, with canonical loss of homeostatic genes and increased expression of APOE, TREM2, CLEC7A, LPL, and SPP1 (33, 34). Human single-nucleus and spatial studies identify partially overlapping, but (49) not identical, plaque-associated, interferon-responsive, lipid-metabolic, and antigen-presenting programs (50, 51). Mouse-derived DAM signatures should therefore be used as comparative reference modules rather than treated as one-to-one human cell identities. Within human tissue, plaque and tau burden, white-matter injury, vascular proximity, and brain region often explain more variation than the diagnostic label alone.

A molecular cluster is descriptive, not intrinsically protective, toxic, exhausted, or senescent. Functional interpretation requires evidence about the initiating stimulus, anatomical niche, substrate handled, metabolic capacity, and consequence for neurons, synapses, or vasculature. A state may express inflammatory genes while retaining effective phagocytosis or repair, whereas a lipid-laden state with modest cytokine expression may be harmful because migration, lysosomal degradation, or cholesterol export is impaired (50, 52).

Risk genes such as APOE, TREM2, CD33, INPP5D, PLCG2, BIN1, and PICALM can alter sensing, endocytic trafficking, signaling thresholds, and metabolic adaptation rather than Aβ production itself (53). Phagocytosis should be evaluated as a sequence comprising chemotaxis, enclosure, internalization, phagosome maturation, lysosomal degradation, and export of metabolic products (54, 55). Increased CD68 or lysosomal-gene expression does not prove efficient clearance and may instead indicate persistent substrate accumulation (56, 57). Likewise, the term senescent should be supported by durable cell-cycle arrest, DNA-damage and chromatin features, and a senescence-associated secretory phenotype; exhausted-like should denote demonstrable loss of effector capacity under sustained stress, not transcriptomic similarity alone (58, 59).

### 4.2 The TREM2-APOE axis: plaque containment, lipid sensing, and metabolic load

TREM2 signals through DAP12/TYROBP-SYK and supports microglial survival, migration, lipid sensing, phagocytosis, and metabolic adaptation (As illustrated in **Figure 2**) (22, 60). Human genetic evidence establishes TREM2 as an AD-risk pathway, and CSF sTREM2 provides an *in-vivo* response marker, but most evidence assigning stage-specific cellular functions comes from amyloid or tau models and human-derived experimental systems. In amyloid-dominant mouse models, intact TREM2 generally supports plaque compaction and limits neuritic exposure; in tau-rich or chronically activated contexts, the consequences depend on timing, dose, metabolic reserve, and the surrounding lesion (60–65). Thus, neither TREM2 activation nor inhibition can be assumed to be uniformly beneficial.

**Figure 2:**
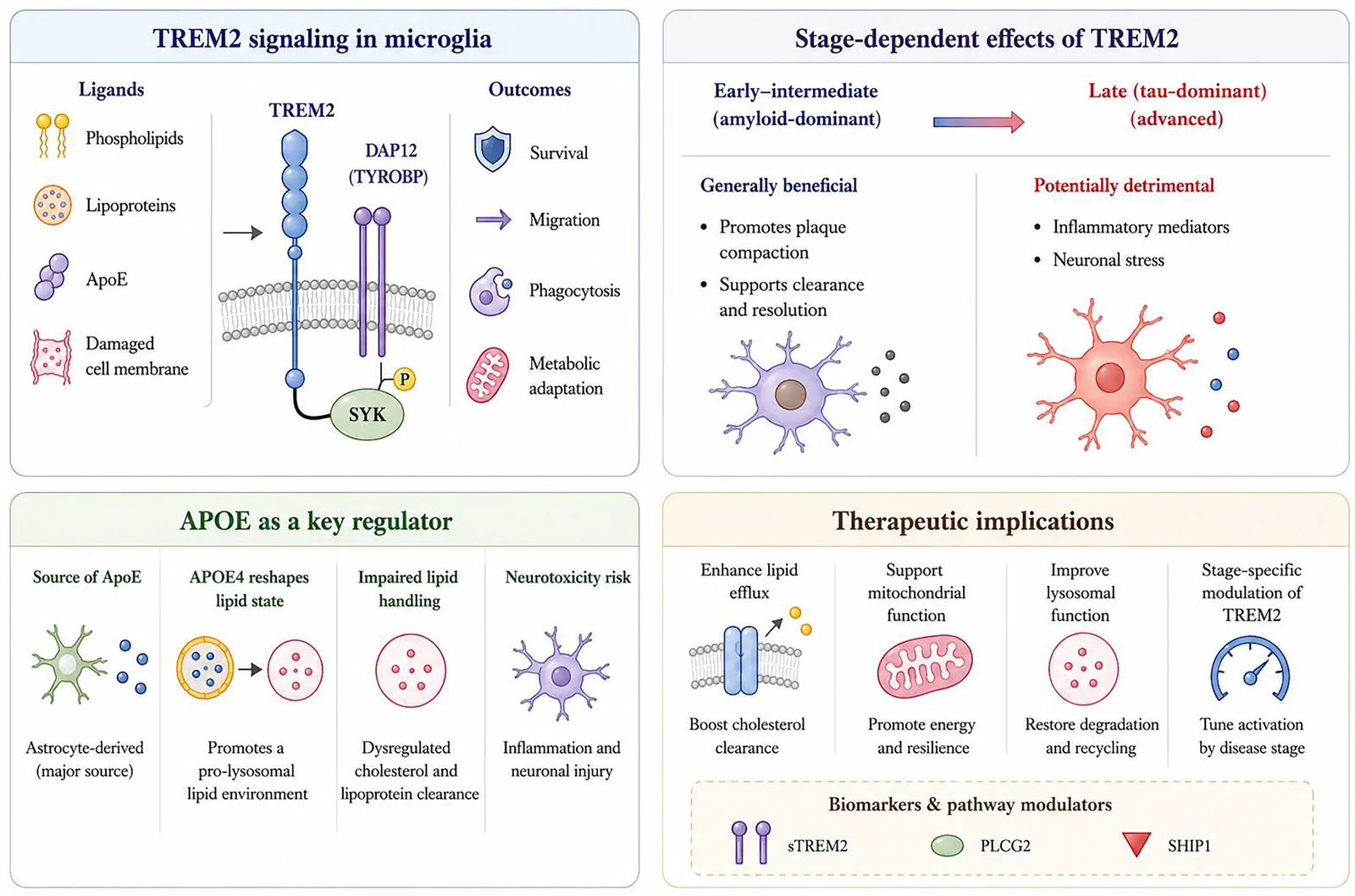
Regulation of microglial plaque responses and metabolic adaptation by the TREM2-APOE axis. Following Aβ deposition, TREM2 signaling through DAP12/TYROBP–SYK promotes microglial migration, phagocytosis, and plaque encapsulation while supporting lipid metabolism and lysosomal function. APOE4 favors intracellular lipid retention and microglial dysfunction. Because the effects of this pathway vary across disease stages, therapeutic targeting should account for pathological context, APOE genotype, and microglial metabolic state.

APOE is a second major node in this transition. Human genetic, neuropathological, imaging, and iPSC studies support effects of APOE4 on glial lipid handling and neurovascular integrity, whereas the direction and cell specificity of individual mechanisms remain partly model-dependent (66, 67). APOE4 can promote poorly lipidated lipoprotein particles and intracellular lipid retention and can modify TGFβ-related checkpoints and sex-dependent peripheral immune interactions. In human APOE4/4 brain tissue, ACSL1-high lipid-droplet-enriched microglia have been linked to neurotoxic lipid release, while selective manipulation of astrocytic or microglial APOE and APOE3 Christchurch has shown context-dependent protection in experimental systems (68–72). These findings support biological plausibility but do not yet define a clinically actionable APOE-specific immune intervention.

Taken together, the TREM2-APOE axis appears to redistribute phagocytic and metabolic demand as pathological load rises (68, 73). Increasing uptake without restoring lysosomal flux, mitochondrial function, and ABCA1/LXR-dependent cholesterol efflux may simply transfer extracellular burden into glial cells. This is an authors’ synthesis derived from convergent model and human observational evidence. It predicts that the effect of TREM2 modulation will depend on whether the dominant limitation is lesion recognition, uptake, degradation, lipid export, or tissue repair, and whether Aβ, tau, vascular injury, or mixed pathology predominates.

TREM2 exists as a membrane receptor and as soluble TREM2 (sTREM2), generated largely through ADAM10/17-mediated shedding (62). CSF sTREM2 changes across the AD continuum and may reflect microglial response, receptor turnover, or shedding; it is not a direct measure of membrane TREM2 signaling or of a specific protective state (62, 71). Its value as a companion diagnostic for TREM2-directed treatment remains experimental.

Downstream checkpoints add further complexity. PLCG2 variants and INPP5D/SHIP1 signaling alter phosphoinositide pathways, but experimental effects depend on developmental stage, lesion context, and the cellular system studied (62, 74). Translational approaches therefore require lesion and cell specificity and clinically demonstrated benefit, not target engagement alone. In the phase 2 AL002 trial in early AD, TREM2 engagement and pharmacodynamic changes did not translate into improvement on the primary clinical endpoint, and MRI abnormalities resembling ARIA were frequent (75). This negative trial reinforces the need to define dose, disease window, vascular risk, and the functional state being modified.

### 4.3 Inflammasomes, nucleic-acid sensing, and immune checkpoints

Mechanistic evidence for NLRP3 activation by lysosomal injury, K+ efflux, mitochondrial reactive oxygen species, and oxidized lipids derives predominantly from cell systems and AD mouse models (76, 77). These studies show that inflammasome activation can produce caspase-1 cleavage and IL-1β/IL-18 release and can alter astrocytic, endothelial, phagocytic, and synaptic outcomes. In human AD, increased inflammasome-related signals and their association with pathology support relevance but do not, by themselves, establish that this pathway is a primary driver in every patient or disease stage.

The cGAS-STING and type I interferon pathways provide a related example. Experimental perturbation links mitochondrial or nuclear DNA sensing to interferon responses and synaptic injury, while transient signaling can also participate in host defense and abnormal-protein processing (77). NLRP3 and tau pathology may reinforce one another in models, but the direction and clinical importance of this circuit in human AD remain incompletely resolved (78, 79). Peripheral metabolic inflammation, infection, and gut-derived signals may modify pathway thresholds.

NF-κB, NLRP3, cGAS-STING, and type I interferon signaling are not independent pathways (76, 77). Leakage of mitochondrial DNA can activate cGAS-STING; STING and TLR signaling provide the transcriptional priming required for NLRP3; and inflammasome-mediated cellular injury releases additional nucleic acids and ATP (35, 77). Checkpoints such as CD33, SIRPα, TIM-3, and TGFβ constrain the transition from homeostatic surveillance to nonselective phagocytosis (80, 81), although excessive restraint may impede Aβ processing.

### 4.4 Synaptic clearance and misrecognition

Synaptic loss correlates more closely with cognitive impairment than plaque count in human AD, but many proposed routes of complement- and microglia-mediated synapse removal were established in developmental or AD mouse models (82–88). Microglia can recognize candidate substrates through CR3, TREM2, MerTK/Axl, SIRPα-CD47, phosphatidylserine, and purinergic receptors. In experimental systems, Aβ oligomers, tau-related stress, altered neuronal activity, and membrane-lipid externalization can increase ‘eat-me’ signals on synapses. Demonstrating the same mechanism in human AD requires spatial evidence that tagged synapses are functional before engulfment, together with cell-specific functional validation (As illustrated in **Figure 3**).

**Figure 3:**
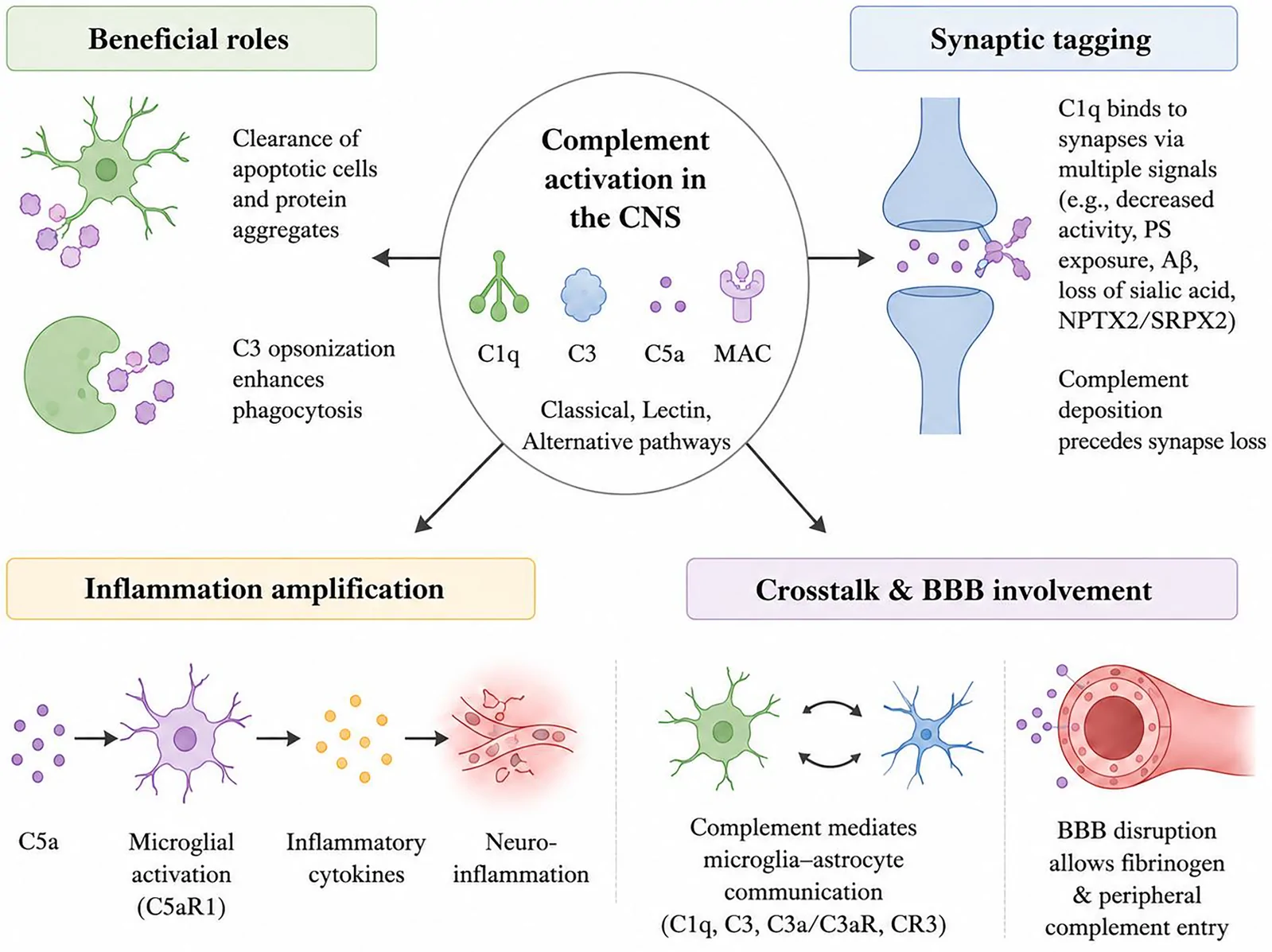
Synaptic clearance and misrecognition in Alzheimer’s disease. Aβ, tau pathology, altered neuronal activity, and oxidative stress promote complement deposition and phosphatidylserine exposure, leading to the aberrant tagging of otherwise functional synapses. Microglia and astrocytes mediate synaptic engulfment through distinct phagocytic pathways. Whereas controlled removal of damaged structures may support tissue repair, persistent or non-selective engulfment contributes to synaptic loss and cognitive decline.

Assessment of phagocytosis must specify the substrate removed and the subsequent tissue consequence (84, 89). Clearance of dead cells, disrupted synapses, myelin debris, or compacted amyloid can support repair, whereas removal of viable synaptic elements can be harmful. The therapeutic priority proposed here—reduce inappropriate synaptic tagging, reinforce protective signals such as CD47-SIRPα, or redirect uptake toward pathological material—therefore represents a mechanistic strategy derived mainly from preclinical evidence, not an established clinical intervention (90–93).

Whether a synapse is engulfed depends not only on complement but also on neuronal activity and glial metabolism (94, 95). Low-activity synapses are more likely to display C1q and phosphatidylserine, whereas highly active neurons can release ATP, chemokines, and exosomes that modify microglial behavior (96, 97).

Astrocytes also phagocytose synapses directly (98–100). MEGF10- and MERTK-dependent astrocytic engulfment supports adult hippocampal circuit homeostasis; in AD, it may remove dystrophic synapses but may also become inefficient because of Aβ-associated dysfunction (101–103). Astrocytic phagocytosis can compensate for inadequate microglial function, whereas chronic inflammatory activation of both cell types may jointly amplify synaptic loss (102, 104). Studies should quantify separately the proportion of synaptic material entering microglial and astrocytic lysosomes and distinguish presynaptic from postsynaptic structures (105).

## 5 Astrocytes: metabolic support, synaptic regulation, and the vascular interface

### 5.1 A continuum of reactive states

Astrocytes ensheath synapses, nodes, and microvessels and serve as an interface for metabolism and information flow in the brain (106–108). Their homeostatic functions include K+ buffering, glutamate uptake, lactate and lipid supply, cholesterol synthesis, synapse formation, regulation of blood flow, and AQP4-dependent water transport (108, 109). In AD, increased GFAP expression is only a superficial indicator of reactivity. Single-cell studies have identified disease-associated, metallothionein-high, oxidative-stress, lipid-metabolic, extracellular-matrix, interferon-responsive, and senescence-like astrocytic states (110, 111). These programs may overlap within the same cell and are closely associated with plaque distance, brain region, APOE genotype, and disease stage (112).

The A1/A2 framework was derived from specific acute-stimulation experiments. The finding that IL-1α, TNF, and C1q can induce a neurotoxic astrocytic program established an important model mechanism of microglia-astrocyte communication, but it does not justify assigning human AD astrocytes to discrete A1 or A2 categories (113, 114). Human single-cell and postmortem studies more often identify overlapping reactive modules whose functional consequences depend on anatomical location, disease stage, and metabolic context. Functional labels should therefore be reserved for astrocyte states with direct perturbation or physiological evidence.

Astrocytes have bidirectional effects on Aβ (115, 116). They contribute to Aβ clearance through LRP1, ApoE, proteases, and lysosomal phagocytosis and form a glial boundary around plaques that may restrict diffusion. Chronic reactivity, however, can increase enzymes involved in APP processing, reduce effective degradation, release inflammatory mediators, and modify Aβ aggregation through ApoE-containing lipoproteins (3, 117, 118). Astrocytes also participate in tau uptake, degradation, and propagation (119). Intracellular tau or tau burden in adjacent neurons may disrupt Ca2+ signaling, mitochondrial function, and neurotransmitter transport, shifting astrocytes from a supportive role toward amplification of network excitability and inflammation (119, 120).

### 5.2 Lipid, glutamate, and mitochondrial homeostasis

During high neuronal activity or oxidative stress, neurons transfer excess fatty acids to astrocytes. If astrocytic mitochondrial oxidation and lipid-droplet buffering are insufficient, oxidized lipids may be released back into the extracellular environment and further induce microglial inflammation (21, 121). Glutamate homeostasis is another critical interface (122). Aβ, inflammatory mediators, and oxidative stress can reduce transporter abundance at the membrane or impair transporter function, resulting in glutamate spillover, excessive NMDA-receptor activation, and increased neuronal Ca2+ load (122). Excitotoxicity can then expose phosphatidylserine on synapses, promote ATP release, and activate complement, attracting microglial phagocytosis (122–124). Senescent astrocytes show reduced proliferative and migratory capacity, secretion of a senescence-associated secretory phenotype (SASP), loss of AQP4 polarity, and impaired metabolic support (125, 126). The neuron-astrocyte lactate shuttle may also be disrupted in AD (108). Lactate generated by excessive glycolysis can participate in histone lactylation and reprogram gene expression in microglia and astrocytes, linking metabolism to immune memory (127). Glutathione and Nrf2-dependent antioxidant systems are central to astrocytic neuroprotection (121). Astrocytes provide cysteine precursors and remove ROS, but these capacities decline in the context of APOE4, ageing, and mitochondrial injury (121). Oxidized lipids and disturbed iron metabolism may further increase susceptibility to ferroptosis (121).

### 5.3 AQP4 polarity, interstitial fluid exchange, and neurovascular coupling

AQP4 is highly enriched in perivascular astrocytic endfeet, and its polarized localization depends on the dystrophin-associated complex and basement-membrane signaling (128, 129). Total AQP4 abundance may increase with AD and ageing, while perivascular localization often declines, impairing the exchange of water and solutes (129). Current evidence indicates that sleep, arterial pulsation, perivascular spaces, and AQP4 localization jointly influence interstitial fluid exchange and protein clearance; AQP4 is not the sole determinant of this process (129, 130). Endfoot function also contributes to neurovascular coupling (128, 131). Astrocytes regulate arteriolar tone through Ca2+-dependent arachidonic-acid metabolites, K+, and adenosine signaling (128, 131). Reactive astrocytes, pericyte injury, and endothelial nitric-oxide imbalance can prevent local blood flow from matching neuronal activity, creating a cycle of hypoperfusion, metabolic stress, and glial activation (128, 131, 132). Vascular-wall Aβ deposition damages smooth-muscle cells and pericytes and narrows basement-membrane drainage pathways; antibody-mediated mobilization of Aβ may transiently increase vascular burden (8, 133). These processes provide a neurovascular explanation for ARIA-E and ARIA-H and indicate that strategies to enhance interstitial clearance must also protect the vessel wall (8, 133).

## 6 Complement and glial-vascular communication

### 6.1 Bidirectional roles of complement in clearance, synaptic tagging, and inflammatory amplification

The classical, alternative, and lectin complement pathways can be initiated within the central nervous system (As illustrated in **Figure 4**) (134). Cell-specific experimental studies identify microglia as a major source of C1q and show that C1q recognition, C3 opsonization, CR3-dependent uptake, C5a signaling, and membrane-attack-complex formation can influence clearance and tissue injury (134–136). In human AD brain and fluid studies, complement components and receptors are associated with plaques, tau pathology, dystrophic neurites, synaptic loss, and cognitive decline; these associations indicate pathway engagement but do not by themselves establish the direction or substrate specificity of complement action.

**Figure 4:**
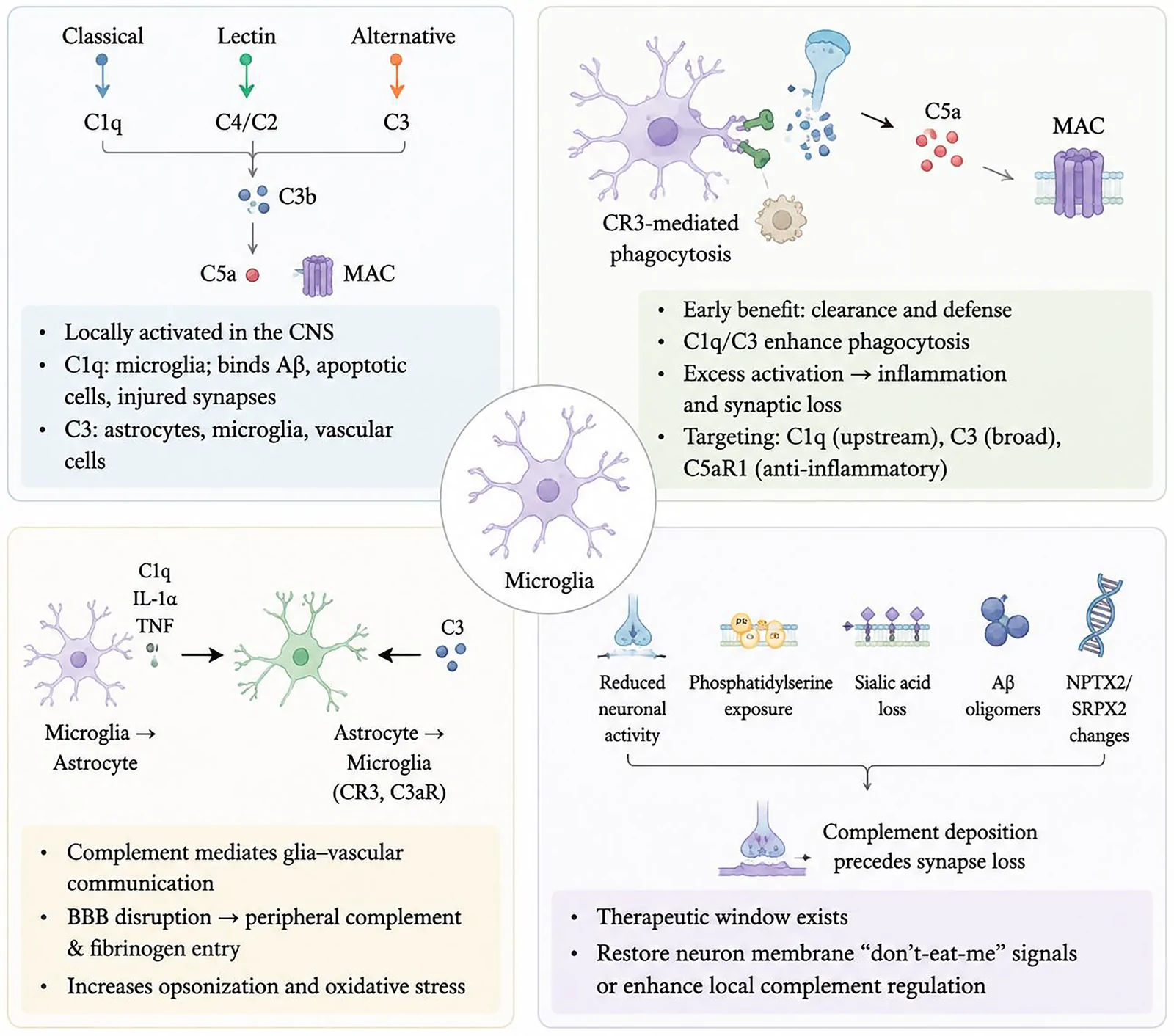
Context-dependent roles of the complement system in Alzheimer’s disease. Complement activation facilitates the clearance of pathological structures through C1q-mediated recognition, C3 opsonization, and CR3-dependent phagocytosis. Sustained activation, however, may promote the elimination of functional synapses and amplify neuroinflammation. These effects vary by disease stage and brain region, indicating that therapeutic strategies should preserve physiological clearance while limiting pathological complement activity.

Complement effects are stage- and context-dependent. Early or spatially restricted activity can facilitate host defense, apoptotic-cell removal, aggregate processing, and tissue repair, whereas sustained or poorly regulated activation can amplify inflammation and tag vulnerable synapses (24, 137). Most causal evidence for these opposing effects comes from genetic or pharmacological manipulation in animal models. Upstream C1q or broad C3 inhibition may also remove physiological functions, whereas C5aR1 antagonism may more selectively reduce inflammatory amplification; none of these approaches has yet demonstrated clinical efficacy in AD (138, 139).

Complement also participates in glial-vascular communication. In experimental systems, microglial IL-1α, TNF, and C1q can reprogram astrocytes, while astrocyte-derived C3 can signal to microglial CR3 and C3aR (113, 114). After BBB disruption, fibrinogen and circulating complement may enter the parenchyma and increase local opsonization and oxidative stress (140, 141). These pathways are biologically plausible in human AD, but their magnitude and temporal order vary with cerebral amyloid angiopathy, vascular leakage, infection, and systemic complement activity.

The mechanism by which C1q selectively marks synapses remains incompletely defined (142). Candidate determinants include reduced neuronal activity, phosphatidylserine exposure, altered sialylation, Aβ binding, and changes in endogenous regulators such as NPTX2 and SRPX2. In mouse models, complement deposition can precede complete synaptic degeneration, suggesting a potential intervention window (142–144). Whether this window can be identified safely in patients is unknown. Local restoration of neuronal ‘do-not-eat-me’ signals or endogenous complement regulation may offer a better balance than systemic blockade, but this remains a preclinical hypothesis.

### 6.2 Key ligand-receptor networks and therapeutic tractability

Microglial-astrocytic communication includes several potentially druggable axes (As summerized in **Table 2**) (145). Microglial IL-1α, TNF, and C1q can induce specific astrocytic programs; astrocytic IL-3 acts through IL-3Rα to enhance microglial migration and Aβ processing; APOE-TREM2/LRP1 links lipid transport to state transition; LGALS3 and integrin signaling influence coordinated glial responses around plaques; and SPP1-CD44/integrin signaling is often enriched in perivascular and highly phagocytic cells (120, 146).

**Table 2:** Major microglia-astrocyte-complement-vascular interaction axes.

Interaction axis	Upstream source	Downstream cell/receptor	Principal effect	Intervention risk
IL-1α/TNF/C1q	Activated microglia	Astrocytes	Induction of specific reactive programs, C3 expression, and metabolic changes	Excessive blockade may weaken injury responses (15, 40, 113)
IL-3	Astrocytes	Microglial IL-3Rα	Enhanced migration and Aβ processing	Appropriate human dose and disease window remain uncertain (146)
APOE-TREM2	Astrocytic/microglial lipoproteins	Microglial TREM2 and lipoprotein receptors	Plaque containment, lipid uptake, and state transition	Sustained activation may increase lipid-droplet and lysosomal burden (22, 73)
C1q-C3-CR3	Microglia/astrocytes/peripheral complement	Microglial CR3	Opsonization and synaptic engulfment	Upstream inhibition may impair physiological clearance (88, 135)
C5a-C5aR1	Complement cascade	Microglia and other myeloid cells	Inflammatory amplification and chemotaxis	Infection risk and immune defense must be considered (138, 151)
Fibrinogen-CD11b	Plasma proteins entering after barrier leakage	Microglial CR3	ROS generation, inflammation, and phagocytic-state induction	Effect depends on the extent of BBB disruption (140, 141)
Cyclophilin A-MMP9	APOE4 pericytes	Endothelium/basement membrane	Degradation of tight junctions and basement membrane	Systemic inhibition may interfere with physiological repair (152)

Chemokine axes govern spatial rearrangement of cells (35, 45). CX3CL1-CX3CR1 supports neuron-microglia homeostasis, CCL2-CCR2 is more closely associated with recruitment of peripheral monocytes, and CXCL10-CXCR3 is linked to interferon responses and T cells (147). Growth factors and checkpoints add another level of control. TGFβ maintains microglial identity and barrier function but may impose excessive checkpoint restraint in an APOE4 context; CSF1 and IL-34 share CSF1R but have distinct spatial sources; VEGF may support vascular repair but also increase permeability; and PDGF-BB maintains pericytes, although excessive or mistimed signaling may drive maladaptive vascular remodeling (148).

The druggability of a receptor depends on cell specificity, ligand redundancy, disease window, and the availability of a pharmacodynamic marker linked to tissue protection (51, 149). Ligand-receptor tools such as CellPhoneDB and NicheNet infer candidate communication from expression patterns; they do not demonstrate ligand release, receptor activation, causal direction, or functional benefit. Candidate axes should be confirmed by spatial colocalization, protein or phosphorylation measurements, receptor blockade and rescue, and cell-specific physiological readouts (150).

## 7 The blood-brain barrier, neurovascular unit, and brain-border immunity

### 7.1 The BBB and neurovascular unit as boundary conditions of the immune microenvironment

The BBB comprises endothelial cells, tight junctions, pericytes, basement membrane, astrocytic endfeet, and perivascular immune cells (As illustrated in **Figure 5**) (141) (As summarized in **Table 3**). Human imaging, postmortem, and single-nucleus studies demonstrate early and regionally variable abnormalities in barrier permeability, endothelial transport, pericyte support, basement membrane, cerebral amyloid angiopathy, and perivascular spaces (153–155). These observations establish vascular involvement in AD but do not imply that all abnormalities share one cause or that barrier leakage alone initiates disease.

**Figure 5:**
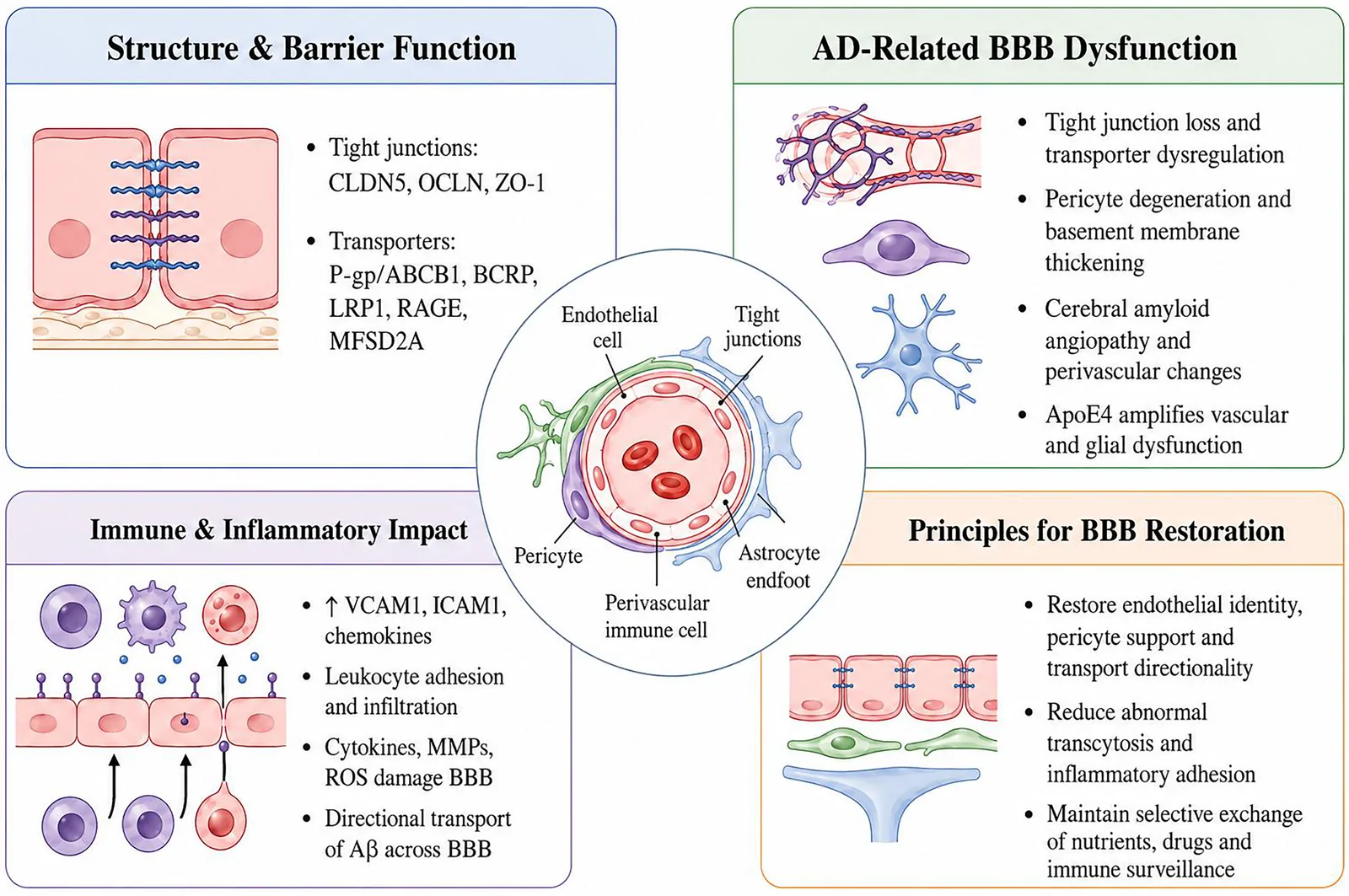
The blood-brain barrier and neurovascular unit shape the brain immune microenvironment in Alzheimer’s disease. In Alzheimer’s disease, APOE4-associated pericyte dysfunction, altered endothelial transport, loss of AQP4 polarization, and inflammatory signaling collectively compromise blood–brain barrier integrity, impair Aβ clearance, and facilitate the entry of peripheral immune components into the brain parenchyma. Therapeutic strategies should therefore aim to restore neurovascular homeostasis and selective transport rather than simply increase barrier tightness.

**Table 3:** AD-related alterations in components of the neurovascular unit.

Neurovascular component	AD-related alteration	Effect on the immune microenvironment	Candidate biomarkers/methods
Endothelial cells	Reduced CLDN5/OCLN, increased VCAM1/ICAM1, and transport reprogramming	Increased leakage and leukocyte adhesion; reduced Aβ efflux	DCE-MRI is clinically available but not validated as an immune companion diagnostic; soluble VCAM1 and endothelial omics are experimental (159)
Pericytes	Reduced PDGFRβ signaling and activation of CypA-MMP9	Basement-membrane injury and impaired capillary regulation and clearance	CSF sPDGFRβ and quantitative pericyte coverage are research measures; no validated clinical cut-off (132)
Astrocytic endfeet	AQP4 depolarization and abnormal Ca2+-blood-flow coupling	Reduced interstitial fluid exchange and local perfusion	Perfusion MRI and sleep assessment are clinically available; AQP4 localization as a stratifier is experimental (129, 130)
Basement membrane/ECM	Altered collagen and laminin; CAA	Changes in cell migration and vascular Aβ deposition	MRI markers of CAA and small-vessel disease are clinically established; matrix proteomics remains experimental (159)
Transport systems	Reduced LRP1/P-gp and relatively increased RAGE	Reduced Aβ clearance and increased influx of peripheral Aβ	Research PET/pharmacokinetic probes and CSF transport ratios; not validated for routine treatment selection (141, 157)
Perivascular immune cells	Enhanced SPP1 and related signaling	Induction of microglial phagocytic states and inflammation	Spatial omics and border-associated macrophage markers are experimental and not measurable routinely in living patients (160)

APOE4 provides a mechanistic bridge between glial and vascular pathology. Human observational studies associate APOE4 with BBB dysfunction and cognitive decline, while human *in-vitro* BBB systems and animal studies implicate pericyte and cyclophilin A-NF-κB-MMP9 signaling (28, 152). Loss of perivascular AQP4 polarization and altered endothelial transport may further impair clearance, but these measures are not yet validated treatment-selection biomarkers.

The consequences of BBB injury extend well beyond entry of blood-derived components into the brain (140). Upregulation of endothelial VCAM1, ICAM1, and chemokines promotes adhesion or entry of T cells, monocytes, and neutrophils (156), while glial release of IL-1β, TNF, VEGF, matrix metalloproteinases (MMPs), and ROS further damages the barrier. Aβ transport across the BBB is directional (157). Pericytes and astrocytes regulate these transporters through secreted factors, indicating that Aβ clearance is a coordinated function of the neurovascular unit rather than an endothelial-cell-autonomous process (157).

BBB repair should not be equated simply with increasing barrier tightness (158). A physiological barrier must restrict nonselective permeability while preserving selective exchange of nutrients, metabolites, drugs, and immune-surveillance signals (158). Excessive reinforcement of junctions may hinder necessary transport and waste clearance. Therapeutic goals should instead include restoration of endothelial identity, pericyte support, and transport polarity, together with reduction of abnormal transcytosis and inflammatory adhesion (158).

### 7.2 Cerebral amyloid angiopathy and ARIA: the vascular cost of immune clearance

Cerebral amyloid angiopathy (CAA) results from Aβ deposition in the walls of leptomeningeal and cortical arterioles and is common in AD, particularly among APOE4 carriers (140). Vascular Aβ can damage smooth-muscle cells and pericytes, impair vascular reactivity, and recruit perivascular macrophages and microglia. Because its clearance is constrained by perivascular drainage and vessel-wall integrity, CAA represents a major vascular vulnerability within the AD brain immune microenvironment (140).

Anti-Aβ monoclonal antibodies can mobilize Aβ and promote immune-mediated clearance. When the clearance load exceeds the processing capacity of the vessel wall and drainage pathways, however, edema, leakage, and microhemorrhage may occur as amyloid-related imaging abnormalities (ARIA-E and ARIA-H) (161–163). APOE4, pre-existing microbleeds, and greater CAA burden all increase ARIA risk (161). ARIA may therefore reflect a mismatch between immune-clearance load and the repair capacity of the neurovascular unit rather than nonspecific drug toxicity alone.

Microglia and perivascular macrophages can remove antibody-coated Aβ, but sustained overload may also release inflammatory mediators and ROS. Complement activation, AQP4 depolarization, and endothelial barrier injury further increase vascular permeability (138, 151, 162). ARIA risk assessment should therefore integrate APOE genotype, microbleeds, CAA burden, and BBB status, while dose optimization, gradual titration, and modulation of immune effector function may reduce vascular injury (158).

A reduction in Aβ does not necessarily coincide with restoration of neurovascular homeostasis (129, 162). Long-term assessment should also include cerebral blood flow, BBB integrity, and glial and vascular function. Treating pathological clearance and tissue repair as consecutive but distinct therapeutic phases may better reflect the biological course of anti-Aβ immunotherapy.

### 7.3 Border-associated macrophages, peripheral immunity, and meningeal lymphatics

Not all myeloid cells in the brain are parenchymal microglia. Macrophages in the meninges, choroid plexus, and perivascular spaces have distinct developmental origins and transcriptional profiles and participate in immune exchange among blood, cerebrospinal fluid, and brain parenchyma (140, 160, 164). In AD, these border-associated macrophages may contribute to vascular Aβ clearance and regulate the states of nearby microglia (140). Distinguishing myeloid populations therefore requires cell-specific markers together with spatial localization rather than reliance on IBA1 or CD68 alone (16, 18).

Peripheral immune cells and brain-border structures jointly shape the AD immune microenvironment. The actions of T cells, neutrophils, and monocytes depend on disease stage, location, APOE genotype, and other factors. Their entry into the central nervous system may result from barrier abnormalities or be actively driven by endothelial adhesion molecules and chemotactic signals (147, 156). Meningeal lymphatic vessels and the choroid plexus further regulate the trafficking of antigens, metabolites, and immune cells; age-related decline in drainage and barrier function may worsen this imbalance (164–166). The dynamic relationships among brain parenchyma, cerebrospinal fluid, meningeal lymphatics, and blood should therefore be incorporated into interpretation of peripheral biomarkers.

Brain-border compartments also provide potential routes for therapeutic delivery (160). Intrathecal administration, focused ultrasound, and intranasal delivery can increase central drug exposure but may also disrupt BBB, vascular, and border-immune homeostasis. Delivery strategies should therefore be evaluated not only for drug exposure but also for their effects on the brain immune microenvironment and neurovascular function.

## 8 Pathological extensions of the immune microenvironment: tau propagation, white-matter injury, and systemic context

### 8.1 Tau pathology and glial interactions

The relationship between tau pathology and the brain immune microenvironment is strongly stage-dependent, but evidence sources should be separated (119). In cell and animal models, microglia and astrocytes can take up extracellular tau; early uptake may limit exposure, whereas incomplete lysosomal degradation can permit re-release. Experimental depletion and perturbation studies show that microglia may either restrain or facilitate tau seeding depending on the presence of amyloid, TREM2 function, and the stage examined (61, 76). Human postmortem and biomarker studies demonstrate coordinated tau and glial changes but cannot determine whether a given state preceded, followed, or merely accompanied neuronal injury.

Astrocytic tau uptake can alter glutamate transport, lipid metabolism, and network support in experimental systems, and APOE4 may amplify these responses (72, 119). The NLRP3-tau feedback loop is supported mainly by model interventions, whereas evidence in patients remains associative. Accordingly, glial uptake should not be equated with effective clearance, and co-occurrence of a glial state with tau burden should not be described as causal without genetic, pharmacological, or rescue evidence.

The local immune and synaptic milieu around Aβ plaques may facilitate tau seeding, and later neocortical spread may coincide with broader glial and synaptic dysfunction (167). This stage sequence is a working model rather than a universal trajectory. Therapeutic studies of the tau-glia axis should therefore preserve potentially useful uptake while testing whether inflammatory feedback or synaptic tagging is reduced. Outcomes should integrate tau burden, target engagement, synaptic integrity, vascular safety, and clinical function rather than rely on a transcriptomic state alone.

### 8.2 White matter, myelin, and oligodendrocytes

AD research has long emphasized grey-matter pathology, but white-matter abnormalities can be detected during the preclinical phase (168, 169). Age-related myelin damage generates large amounts of cholesterol-rich debris, increasing the phagocytic and metabolic load on microglia and astrocytes. White-matter injury therefore not only impairs neural conduction but also reflects disruption of brain immune and lipid homeostasis.

Microglial clearance of myelin debris supports remyelination, but impaired cholesterol efflux can weaken this reparative function (170–172). Astrocytes regulate myelin repair bidirectionally through lipid support and inflammatory signaling (168, 169, 173). APOE4 further disrupts glial lipid processing and myelin renewal, indicating that abnormal lipid metabolism is a shared pathological link among multiple glial cell types (67).

White matter is also highly susceptible to hypoperfusion and vascular injury. BBB leakage, pericyte dysfunction, and chronic ischemia can aggravate myelin damage and sustain glial activation (132, 159). Incorporating white matter into the brain immune-microenvironment framework helps integrate glial phagocytosis, lipid metabolism, myelin injury, and neurovascular function and may broaden the range of therapeutic targets in AD (168).

### 8.3 Modulation by ageing, infection, and systemic immunity

Age should not be treated as merely a covariate in AD research; it is a major biological context that reshapes the brain immune microenvironment. Ageing can simultaneously impair microglial clearance, astrocytic metabolic support, and AQP4 polarity, while reducing endothelial and pericyte function and increasing complement activity (30, 31). Cellular senescence should be distinguished from general age-related functional decline. It is characterized by stable cell-cycle arrest, DNA damage, and a senescence-associated secretory phenotype (SASP); senescent glial and vascular cells may further aggravate chronic inflammation, clearance failure, and BBB injury (31, 125).

Systemic inflammation can amplify these changes. Infection, metabolic abnormalities, and chronic disease may modulate brain immune responses through endothelial activation and myeloid immune remodeling (174). The gut microbiome and infection history may also influence glial states, but current evidence more strongly supports their role as disease modifiers than as universal direct causes of AD (175).

## 9 Human evidence and causal validation: omics and model systems

### 9.1 Single-cell and spatial omics and human-derived models

Single-cell and single-nucleus RNA sequencing has revealed substantial glial heterogeneity in human AD, but these data are vulnerable to postmortem interval, tissue-processing effects, nuclear-RNA bias, low-abundance transcript loss, and cohort composition (5, 6, 176). Spatial transcriptomics and proteomics retain relationships to plaques, vessels, white matter, and regional pathology, yet spatial proximity and correlated expression remain observational.

Human iPSC systems, organoids, and xenografted human microglia permit isogenic manipulation of APOE, TREM2, and other risk pathways and can supply intervention evidence not obtainable from postmortem tissue (177–179). Their limitations differ: iPSC-derived cells may be immature, organoids lack complete vascular and immune architecture, and chimeric models place human cells in a mouse tissue environment. Findings from these systems should be cross-validated in independent human tissue and longitudinal biomarker cohorts.

We apply a practical evidence hierarchy throughout this review. Human genetics, randomized clinical interventions, and replicated human pathological or biomarker findings receive the greatest translational weight, while cell-specific perturbation and rescue studies provide the strongest mechanistic evidence. Human observational omics defines relevant states and niches but cannot establish direction; animal and *in-vitro* studies establish experimental causality within their models but require species- and context-aware translation. Longitudinal studies linking blood and CSF biomarkers, PET, MRI, cognition, and eventual tissue analysis remain a critical gap (179).

### 9.2 From cell clusters to tissue niches and longitudinal states

Human anti-Aβ immunotherapy studies show that pathological clearance can remodel plaque-associated microglia, providing uncommon *in-vivo* evidence that treatment changes a local immune niche (162). Human spatial proteomics further demonstrates that RNA, surface protein, morphology, and location do not always coincide (51). Nevertheless, these studies do not validate mouse DAM signatures as pharmacodynamic endpoints. Candidate human endpoints should be tied to central target engagement and a tissue-protection measure rather than to expression of a state marker alone.

Studies of TIM-3, APOE-related lipid pathways, and APOE3 Christchurch indicate that target effects depend jointly on cellular source, lipid state, and lesion location (180). These mechanistic observations require validation in independent human brain cohorts, longitudinal biomarker studies, and multiple human-derived models. Given confounding by mixed pathology, ancestry, and prior treatment, human studies should also stratify by Aβ/tau stage, cerebrovascular burden, APOE genotype, sex, and brain region to distinguish AD-specific changes from comorbid or end-stage responses (29).

### 9.3 Research standards and reproducibility

Animal studies should report model type, age, sex, APOE background, disease stage, intervention timing, and cell-targeting specificity and should interpret behavioral results together with pathological, synaptic, vascular, and physiological measures. Human brain studies should control for pathological stage, postmortem interval, RNA quality, age, sex, ancestry, cerebrovascular burden, mixed pathology, medication exposure, and lesion location. These variables should be incorporated into design and stratification.

New cellular states should be described using reproducible molecular modules and spatial localization before functional terminology is assigned. Protective, neurotoxic, exhausted, or senescent labels require cell-specific perturbation or rescue and a defined functional readout (50, 52). Reliable target validation should integrate human relevance, cellular localization, experimental causality, pharmacodynamic engagement, and tissue protection.

## 10 From mechanism to the clinic: strategies targeting the brain immune microenvironment

### 10.1 The anti-inflammatory paradox and principles of intervention

AD-associated immune responses participate in pathological clearance and tissue repair but can also contribute to synaptic and vascular injury. Clinical experience argues against treating this complexity as a single inflammatory axis. The key lies in determining which cells, pathways, substrates, anatomical sites, and disease windows should be targeted, as well as whether the intervention measures can preserve the antibacterial defense and tissue repair functions.

Several negative or inconclusive trials define this translational boundary. Prednisone did not slow clinical progression, and rofecoxib or naproxen did not improve outcomes in established AD (181, 182). In the ADAPT prevention trial, naproxen and celecoxib did not improve cognition, while two years of minocycline did not slow cognitive or functional decline in mild AD (183, 184). More recently, the TREM2 agonist AL002 achieved central target engagement but did not meet its primary clinical endpoint, and MRI changes resembling ARIA were the most frequent treatment-emergent adverse events (75). Collectively, these results show that nonspecific systemic suppression, late intervention, or target engagement without tissue protection is insufficient.

Because immune states differ between lesion-adjacent and distant tissue, interventions also require spatial and state selectivity (51). Targets can act at pathological input, inflammatory amplification, substrate selection, metabolic processing, or tissue repair. A clinically credible strategy should specify the intended state transition, the measurable marker of central engagement, the tissue-protection endpoint, the expected treatment window, and the stopping rule for vascular or infectious toxicity.

### 10.2 Major therapeutic directions and translational boundaries

The aim of brain immune therapy should be to correct a defined functional mismatch rather than chronically deplete or broadly suppress immune cells. TREM2, INPP5D/SHIP1, NLRP3, cGAS-STING, complement, lipid-efflux, astrocytic, and vascular targets differ markedly in evidence maturity. Most have genetic, human observational, human-derived model, or animal support but no established clinical efficacy in AD; AL002 is a notable negative clinical test of TREM2 agonism (75). Therapeutic claims should therefore remain proportional to the development stage summarized in **Table 4**.

**Table 4:** Strategies targeting the brain immune microenvironment and major translational considerations.

Therapeutic direction	Expected benefit	Potential treatment window	Principal risks/reasons for failure	Evidence level/development stage	Suggested companion biomarkers
TREM2 agonism or checkpoint modulation	Enhanced plaque containment and phagocytosis	Preclinical/early, Aβ-dominant disease	Stage- and dose-dependent effects; possible worsening of inflammation in late disease	Human genetics and biomarkers; extensive preclinical work; phase 2 AL002 negative. No established clinical benefit (75)	sTREM2, amyloid/tau status, APOE genotype, MRI vascular safety; microglial PET/lipidomics experimental
NLRP3/cGAS-STING inhibition	Reduced chronic inflammation and interferon programs	High-inflammatory states and tau-propagation phase	Impaired antimicrobial defense and patient heterogeneity	Predominantly cell and animal evidence; hypothesis-generating for AD trials	IL-1β/interferon signatures and tau burden; no validated companion diagnostic
LXR/ABCA1 activation and lipid efflux	Reduced glial lipid-droplet and lysosomal burden	APOE4 or lipid-dysregulated subgroups	Increased peripheral lipogenesis and inadequate CNS exposure	Human-derived cell and animal evidence; no validated AD clinical efficacy	ApoE lipidation and CSF lipidomics; experimental
C5aR1/complement modulation	Reduced inflammation and inappropriate synaptic engulfment	High-complement states with accelerating synaptic loss	Interference with clearance and antimicrobial defense	Strong preclinical mechanism; human observational support; no proven AD treatment efficacy	C1q/C3 fragments and SV2A PET; experimental for treatment selection
GLT-1/AQP4 and astrocytic homeostasis	Reduced excitotoxicity and improved fluid exchange	Early-to-middle-stage network dysfunction	Restored expression may not restore perivascular localization	Preclinical and human observational evidence; candidate targets remain experimental	MRS glutamate, perfusion MRI; AQP4 polarity experimental
BBB/pericyte repair	Reduced leakage and entry of peripheral inflammation	APOE4, CAA, or vascular high-risk subgroups	Altered drug entry and vascular reactivity	Human observational and experimental-model evidence; repair strategies not clinically validated	DCE-MRI, microbleeds/CAA, sPDGFRβ; only MRI measures are clinically established
Combination strategies with anti-Aβ immunotherapy	Reduction of pathological substrate with support for post-clearance repair	Aβ-positive early AD	ARIA, microbleeds, and post-clearance glial metabolic burden	Anti-Aβ therapy has clinical evidence; adjunct immune/repair sequence is hypothesis-generating	Amyloid PET/CSF, MRI ARIA monitoring, GFAP, vascular markers

Anti-Aβ immunotherapy illustrates both the potential and the cost of immune-mediated clearance. Clinical trials demonstrate amyloid reduction and modest slowing of decline in selected early-AD populations, while ARIA and vascular injury show that the rate of clearance can exceed glial and vessel-wall capacity (133, 161, 163). The sequential combination approach proposed here—reduce pathological substrate, limit maladaptive amplification, and support metabolic, synaptic, and vascular repair—is a testable therapeutic hypothesis.

### 10.3 Biomarkers and clinical-trial design

Clinical implementation should begin with measures that are already validated for AD diagnosis or safety monitoring. Disease stage can be defined by amyloid PET or CSF Aβ42/40 and by tau biomarkers, including CSF or plasma phosphorylated tau and tau PET, interpreted with structural MRI, cognition, and functional assessment (185). APOE genotyping, MRI assessment of microbleeds and cerebral amyloid angiopathy, and routine cerebrovascular imaging are clinically available and directly relevant to treatment risk. These measures identify pathology and vascular vulnerability; they do not directly identify a microglial or astrocytic functional state.

Immune and cellular-state measures remain less mature. Blood or CSF GFAP is reproducible and clinically interpretable as an astroglial-injury marker, but it is not state-specific. CSF sTREM2, complement fragments, soluble PDGFRβ, VCAM1, ANGPT2, lipidomic signatures, extracellular vesicles, TSPO or other microglial PET ligands, SV2A PET as a synaptic readout, and spatial or single-cell omics are experimental as treatment-selection tools. No current biomarker can resolve a molecularly defined cell state in a specific living brain region with sufficient specificity to serve as a validated companion diagnostic.

Accordingly, near-term trials should use a layered design: validated Aβ/tau and clinical staging; region-specific MRI or PET for tissue burden and vascular safety; an experimental immune marker for target engagement; and a tissue-protection endpoint such as synaptic integrity, neurodegeneration, perfusion, or cognition. Biomarkers should be labeled as established, clinically available but unvalidated for immune stratification, or experimental. This distinction prevents exploratory measures from being presented as ready for routine clinical decision-making.

### 10.4 A precision-stratification framework based on disease stage × brain region × genotype × cellular state

The proposed disease stage × brain region × genotype × cellular state framework is intended as a research and trial-design matrix. Disease stage can be measured with validated Aβ/tau biomarkers and clinical assessments; brain region with structural MRI, amyloid or tau PET, perfusion or permeability imaging, and regional symptom patterns; genotype with APOE and, in research settings, other risk variants. Cellular state is the least directly measurable dimension and currently requires convergent inference from fluid markers, imaging, and tissue or human-derived models.

The framework should also include age, sex, ancestry, cerebrovascular burden, cerebral amyloid angiopathy, mixed neurodegenerative pathology, systemic inflammatory or metabolic disease, and prior treatment as modifiers. For example, early Aβ-positive disease with low tau burden may prioritize pathological clearance and vascular protection, whereas combined Aβ/tau pathology with complement or glial abnormalities may justify testing anti-amplification or metabolic approaches.

At the research level, immune (I) and vascular (V) dimensions could be studied alongside A/T/N to test whether they improve prognostic or treatment-response stratification (179, 186). We emphasize that A/T/N+I/V is a conceptual proposal. Before clinical adoption, candidate I and V measures require analytical validation, prespecified thresholds, longitudinal reproducibility, demonstration of added value beyond A/T/N and clinical variables, and evidence that biomarker-guided treatment improves outcomes.

## 11 Key unresolved questions and research priorities

Future work should distinguish cell states that directly modify AD progression from compensation, comorbidity, and terminal-stage responses. Establishing causal order will require temporally controlled and cell-specific perturbation, rescue experiments, longitudinal human biomarkers, and multiregional tissue analysis. A second challenge is to convert spatial heterogeneity into clinically measurable signals. Candidate protein panels, extracellular vesicles, imaging ligands, and regional physiological measures need prospective validation against specific cellular states and outcomes. Targets should be tested across disease stage, brain region, APOE genotype, age, sex, ancestry, cerebrovascular disease, and mixed-pathology contexts, with long-term assessment of infection, vascular injury, and off-target synaptic effects.

## 12 Conclusion

The brain immune microenvironment of AD is composed of microglia, astrocytes, complement system, neurovascular units, and the brain marginal zone. These components continuously interact with each other through pathological protein processing, lipid metabolism, synaptic regulation, and vascular function. Its notable features include continuous changes in cell state, spatial heterogeneity, and dynamic remodeling during the disease process. When the pathological load exceeds the capacity for phagocytosis, degradation, lipid processing, and barrier repair, the initial compensatory response may gradually evolve into non-adaptive synaptic clearance, inflammation amplification, and neurovascular dysfunction. Therefore, brain immune therapy for AD should shift from broad anti-inflammatory treatment to functional regulation based on stages and cell states, in order to achieve effective pathological clearance, reduce adverse immune damage, and restore glial cell metabolism and neurovascular homeostasis. Extensive anti-inflammatory treatment and negative trials of TREM2 agonists further indicate that mere biological rationality and target activation are not sufficient. Stratifying patients based on disease stage, brain region, genotype, and cell state, and combining multimodal biomarkers to confirm the activation of central targets and tissue protection will be the key to achieving precise transformation.
